# Psychometric properties and post-hoc CAT analysis of the pediatric PROMIS® item banks anxiety and depressive symptoms in a combined Swedish Child and Adolescent Psychiatry and School sample

**DOI:** 10.1007/s11136-025-03898-y

**Published:** 2025-01-30

**Authors:** Ida Blomqvist, John Eric Chaplin, Eva Henje, Inga Dennhag

**Affiliations:** 1 Department of Clinical Science, Child- and Adolescent Psychiatry, Umeå University, 90185 Umeå, Sweden; 2 Department of Pediatrics, Institute of Clinical Sciences, University of Gothenburg, Gothenburg, Sweden

**Keywords:** Child- and adolescent psychiatry, Depressive symptoms, Item response theory, Graded response model, Computer adaptive testing, Differential item functioning

## Abstract

**Purpose:**

The objective of this study is to assess the psychometric properties and reliability of the Swedish Patient-Reported Outcomes Measurement Information System (PROMIS) item banks for anxiety and depressive symptoms with item response theory analysis and post-hoc computerized adaptive testing in a combined Swedish Child and Adolescent Psychiatry (CAP) and school sample.

**Methods:**

Participants (n = 928, age 12–20) were recruited from junior and high schools and Child and Adolescent Psychiatry Clinics in the region of Västerbotten. Unidimensionality, local independence, and monotonicity was tested. We fitted a graded response model to the data and tested differential item functioning (DIF) for sex, age group, sample type, and language (Swedish vs. U.S.). Moreover, a post-hoc computer adaptive testing (CAT) simulation was performed. All analysis were made in R.

**Results:**

Unidimensionality, local independence, and monotonicity were acceptable. The graded response model yielded acceptable item fit, discriminative, and threshold values for all items in both item banks. DIF for language (Swedish vs. U.S.) was found for two items from the anxiety and one item from the depressive symptoms item banks. A Stocking-lord transformation was used for the items displaying language DIF, and post-hoc CAT simulations were performed. The post-hoc CAT simulation showed reliability around 0.9 for both Swedish and official U.S. item parameters T-scores calibration from within normal limits to severe anxiety and depressive symptoms.

**Conclusion:**

The Swedish pediatric PROMIS item banks of anxiety and depressive symptoms are appropriate to assess mild to severe symptoms of anxiety and depressive symptoms in Swedish school- and CAP samples.

**Supplementary Information:**

The online version contains supplementary material available at 10.1007/s11136-025-03898-y.

## Introduction

The Patient-Reported Outcomes Measurement Information System (PROMIS) was initiated and funded by the National Institutes of Health (NIH) [1] to advance the science of patient-reported outcomes. The PROMIS pediatric project has specifically focused on developing measures for children and youth in the age range of 8 to 17 years across several health domains [1–6]. PROMIS has been developed based on Item Response Theory (IRT) to create item banks, which are calibrated items intended to measure the same underlying construct [2]. IRT-validated item banks open the possibility of using computer adaptive testing (CAT) [7]. CAT presents questionnaire items so that every item uniquely adapts to the respondent based on previous responses. This method iteratively selects the most relevant items. When a stopping rule, e.g., a predefined standard error of measurement, has been reached, no more items are presented [3]. An advantage of CAT is that fewer items must be administered while high test precision is maintained [8].

The pediatric PROMIS item banks for anxiety and depressive symptoms were initially calibrated in a school-based and hospital outpatient sample in the US [4, 5]. These item banks showed promising psychometric evidence [4, 5]. Further studies have demonstrated convergent and discriminant validity among patients with traumatic brain injury [9]. The item banks have been translated and tested among school- and outpatient samples in Brazil [10] and in a Dutch juvenile rheumatoid arthritis sample as well as a general sample [11, 12]. Furthermore, short forms of the anxiety and depressive symptoms item banks have been validated among pediatric cancer patients in China [13]. These studies provide preliminary evidence for good psychometric properties of the pediatric PROMIS item banks of anxiety and depressive symptoms.

Most patient-reported measures in Sweden used clinically and in research, are validated using Classical Test Theory (CTT) [14]. CTT has limitations, such as offering a single standard error of measurement for the total scale regardless of the respondents' ability estimate [15], and being sample-dependent, limiting generalizability. A 2009 Swedish report [14] identified over 100 self-report instruments in Child and Adolescent Psychiatry (CAP) clinics, with more than half lacking validation even in original versions, which is problematic since that limits generalizability.

The current project is a part of the Swedish PROMIS initiative in which several item banks have been translated into Swedish [16–18]. The aim is to implement and establish PROMIS item banks in the Swedish healthcare system. The objective of this present study was to assess the psychometric properties and reliability of the PROMIS item banks for anxiety and depressive symptoms with item response theory analysis and post-hoc computerized adaptive testing in a combined Swedish Child and Adolescent Psychiatry (CAP) and school sample.

## Methods

### Participants

We recruited participants in a school sample from four junior and high schools in different socioeconomic areas in the region of Västerbotten in northern Sweden. This convenience sample included natural science, social science, media, and the arts students. Participants in the clinical sample were recruited from Child and Adolescent Psychiatry (CAP) clinics in corresponding areas in northern Sweden. The patient sample was recruited through flyers and staff working at the CAP clinics. Our previous publications describe the samples [17, 19]. Power calculations were based on EFPAs (European Federation of Psychologists’ Associations AISBL) recommendations of a sample of 700 participants for a 3-parameter study [20].

We recruited respondents between the ages of 12 and 20. The exclusion criteria were 1. non-fluency in written Swedish and 2. inability to complete online or paper forms (e.g., severe dyslexia or psychosis). Sweden's General Data Protection Regulation (GDPR) occurred during the data collection procedure. The original online platform used for student data collection did not comply with the new regulations and could not be used for patient data collection. Therefore, paper forms were used for the patient sample until a proper GDPR-compatible platform was established.

### Measures

The PROMIS Pediatric Item Bank v2.0—Anxiety consists of 15 questions, and the PROMIS Pediatric Item Bank v2.0—Depressive Symptoms consists of 14 questions. They are all based on a five-point response option, ranging from 'never' to 'almost always,' and use a seven-day recall period [4, 5]. The symptom severity of the respondents is given in theta ($\theta ).$ PROMIS provides a formula to transform thetas to T-scores through the equation ($\theta$ * 10) + 50 = T-score. Higher values are operationalized as higher levels of the underlying construct, i.e., higher levels of anxiety or depressive symptoms.

### Procedure

The Regional Ethics Board at Umeå University approved the study. Permission was granted by the PROMIS organization to translate the item banks into Swedish. Our previous publication describes the procedure [16, 17]. The principals of each school permitted the gathering of student data. The class teachers informed the students about the study. We obtained informed oral and written consent from all students on the date of the self-report assessment and parental consent for students 15 years or younger. The data collection for the school sample took place during 2018 and 2019. The Clinical Director of the CAP clinics permitted patient data collection, and clinicians were informed about the study to help with the recruitment. Research assistants obtained consent and informed patients and their parents about the study. Parental consent from patients 18 years or younger was also collected. The respondents received a gift card after completing the questionnaires á 200 SEK.

### Statistical analyses

We conducted data analysis following the suggested methods by Reeve et al. [21, 22], e.g., we analyzed the data with descriptive statistics and checked items for zero frequencies.

Before applying the IRT model, we evaluated its assumptions: unidimensionality, local independence, and monotonicity. To assess unidimensionality, which tests whether the scale measures a single construct, we conducted a single-factor Confirmatory Factor Analysis (CFA) using the following fit indices: the scaled comparative fit index, CFI > 0.95; the scaled Tucker-Lewis index, TLI > 0.95; the scaled root mean square error of approximation, RMSEA < 0.06; and the standardized root mean square residual, SRMR < 0.08 [23]. If the CFA model fit was insufficient, further analyses were performed to ensure unidimensionality, including the Kaiser–Meyer–Olkin (KMO) test, parallel analysis, and Exploratory Factor Analysis (EFA). We randomly split the sample into two equal parts to avoid performing the EFA and CFA on the same sample [24]. We performed the KMO, parallel analysis, and EFA in the first half and the CFA in the second half. The KMO, parallel analysis, and EFA were performed using the R package psych [25]. We conducted the EFA analysis based on the polychoric correlations matrix using the weighted least square (WLS) estimation method. The CFA model fitted the polychoric correlations matrix using the diagonal weighted least square estimator (DWLS) using the R package lavaan [26]. For the KMO, values above 0.90 have been characterized as excellent, above 0.70 as moderate, and less than 0.5 as unacceptable [27]. In the EFA, unidimensionality was assumed when the first factor accounted for at least 20% of the variability, and the ratio of the variance explained by the first to the second factor was greater than four [21, 22, 28].

Secondly, we examined local independence by evaluating the residual correlations after controlling for the dominant factor in the CFA. Residual correlations > 0.20 were considered indicators of local dependence [21, 22].

Monotonicity, the likelihood of respondents selecting higher response categories as their underlying trait level increases, was assessed using nonparametric item response theory with the Mokken package in R [29]. Monotonicity was evaluated with the scalability coefficient h over 0.30 for items and over 0.50 for the item banks [29].

After confirming the IRT model assumptions, we fitted a Graded Response Model (GRM) model appropriate for ordinal response categories using the mirt package in R [30]. The GRM yields the slope and threshold values of the items. The discriminative ability of the item equals the item slope, and a higher value indicates a better discriminative ability. The item thresholds refer to the item difficulty, and for a 5-point option scale, four thresholds are located along with the measured trait. We evaluated the fit of the items with Orlando and Thissen's S-X^2^ statistics, where a non-significant value is an indication of adequate fit (p > 0.001) [31].

We assessed measurement invariance to ensure the measure is equivalent between groups, with differential item functioning (DIF). DIF was evaluated with ordinal logistic regression using the Lordif package in R [32]. A McFadden's pseudo-R^2^ change of 2% was used as a critical value to flag for DIF [32]. We tested DIF for sex (girls vs. boys), age groups (12–15 years vs. 16–20 years), type of sample (school vs. patient sample), and language (Swedish vs. U.S.). Language DIF was tested with the PROMIS1 Pediatric Supplement, downloaded from Datavers [33]. In order to match with the Swedish data, only respondents over 12 years old were kept. Further, respondents that had answered four items or more on the anxiety and depressive item banks, respectively, where kept, resulting in a data set of N = 1510 (54.2% female, mean age 14.26 (SD 1.70)) [32].

The IRT framework conceptualizes reliability with information (I) or the inversely related standard error (SE). Information (I) is inversely related to the standard error (SE), given the underlying ability of the latent construct, i.e., theta ($\theta$) by the equation SE($\theta$) = $1/\sqrt{I(\theta )}$. Information (I) and, subsequently, the standard error (SE) can differ across the continuum of theta. Theta is estimated based on the GRM model and ranges from approximately − 4 to 4. A standard error of 0.548 corresponds to a reliability of 0.70, and a standard error of 0.316 corresponds to a reliability of 0.90 through the equation: reliability = 1 − SE^2^ [34]. We calculated the Swedish sample T-score means using the universal US PROMIS T-score metric via the Health Measures Scoring Centre. For known-group analysis, we compared CAP and school sample T-scores derived from the GRM model. We used an independent sample t-test to assess mean differences between the CAP and school samples and to calculate the effect size, Cohen's d. For Cohen's d, a value of 0.2 is considered small, 0.5 medium, and 0.8 large [35]. Further, we examined floor/ceiling effects by calculating the percentages of participants scoring the minimum (floor) and maximum (ceiling) possible scores. Important floor/ceiling effects were defined as more than 15% of participants achieving the lowest or highest score, respectively [36].

#### Post-hoc computer adaptive testing (CAT)

We used a standard error of 0.316 as the stopping rule, corresponding to a reliability of 0.9. We followed PROMIS practice and used the item with the highest information value for the average level of participation in the population (theta = 0) as the starting item. The catR package in R was used for the simulations [37]. The maximum Fisher Information criterion was used for item selection and expected a posteriori (EAP) estimation to estimate thetas.

To avoid overfitting by performing GRM and post-hoc CAT on the same sample, we randomly divided the sample into an evaluation and a validation sample; this ensures that the post-hoc CAT results are not biased using the same data for parameter estimation as in the CAT. The evaluation sample, slightly under 80% of the total sample, was used for the GRM and gave the calibration item parameters for the post-hoc CAT. We used the validation sample as the response matrix in the post-hoc CAT simulation. We conducted two post-hoc CAT simulations, the first with GRM item parameters from the Swedish sample (i.e., the evaluation sample GRM parameters) and the second with (hybrid) U.S. item parameters obtained from HealthMeasures in order to standardize the PROMIS T-score metric with the U.S. reference sample [38]. For language DIF items (US vs Swedish), the Stocking-Lord method was applied. This method links unique item parameters from one group (e.g., the Swedish sample) to a common metric (the universal US PROMIS T-score metric) [38]. Hence, in the second post-hoc CAT simulation a hybrid approach was used: in the post-hoc CAT, US parameters were applied for all items except DIF language items, which used Stocking-Lord transformed Swedish parameters.

## Results

A total of n = 637 students (mean age 15.73 (SD = 1.76), 61.1% female, 38.9% male) and n = 291 patients (mean age 15.64 (SD = 1.61), 71.4% female, 28.6% male) participated in the study. Missing values were 3.4% for the anxiety item bank and 3.0% for the depressive symptoms item bank. Since both item banks had missing values under 5%, we used listwise deletion per item bank (n = 897, n = 901, anxiety, and depressive symptoms item bank, respectively).

### IRT assumptions

In the CFA, RMSEA exceeded the cut-off for both item banks. However, SRMRs were below the cut-off, and CFI and TLI values exceeded 0.95, indicating an acceptable fit [39]. KMO, parallel analysis, and EFA also supported unidimensionality (see Table 1). No item pairs in either item bank showed local dependence. Monotonicity was shown for both item banks, with item bank coefficient h above 0.5 for both item banks and coefficient h values above 0.3 for all items in both item banks. In summary, the assumptions of unidimensionality, monotonicity, and local independence were considered to be met for the anxiety and depressive symptoms item banks.

**Table 1:** Item response theory (IRT) assumptions analysis of the Swedish pediatric Patient-Reported Outcomes Measurement Information System (PROMIS) Anxiety and Depressive symptoms item banks

	Anxiety	Depressive symptoms
Unidimensionality		
KMO		
Item range	0.92 (3977R1r)–0.98 (953R1r)	0.94 (5041R1r, 5035R1r, 228R1r)–0.97 (488R1r, 3952aR2r, 5047R1r, 679aR2r, 7010)
Item bank	0.95	0.96
Parallel analysis		
Eigen value of factor	[1] 9.85 [2] 0.41 (ratio 24.0)	[1] 10.53 [2] 0.27 (ratio 39.0)
EFA		
Eigen value, proportion variance	9.85, 0.66	10.54, 0.75
Factor score range	0.68 (2230R1r)–0.89 (713R1r, 227bR1r)	0.73 (5047R1r)–0.92 (228R1r)
CFA	CFI = 0.96, TLI = 0.95, RMSEA = 0.10 CI [0.09, 0.11], SRMR = 0.06	CFI = 0.98, TLI = 0.98, RMSEA = 0.11 CI [0.10, 0.12], SRMR = 0.04
Local independence		
Residual correlation (> 0.20) after controlling for dominant factor in CFA	No item pairs > 0.2, no local dependence	No item pairs > 0.2, no local dependence
Monotonicity		
Coefficient h—items	0.42 (2230R1r)–0.65 (713R1r)	0.59 (5047R1r)–0.75 (228R1r)
Coefficient h—item bank	0.55 (0.02)	0.69 (0.01)

*KMO* Kaiser–Meyer–Olkin Factor Adequacy, *EFA* Exploratory Factor Analysis, *CFA* Confirmatory Factor Analysis, *LD* Local Independence, *CFI* Comparative Fit Index, *TLI* Tucker-Lewis Index, *RMSEA* Root Mean Square Error of Approximation, *SRMR* Standardized Root Mean Square Residual

The sample was divided into evaluation and validation samples. The evaluation samples had n = 701 and 702 responses, and the validation samples had n = 196 and 199 responses for the anxiety and depressive symptoms item banks, respectively. We applied a GRM model to the evaluation samples. Table 2 presents the discriminative, threshold, and item fit values of the GRM model for the anxiety and depressive symptoms item banks. All items had acceptable discriminative values ranging from 1.49 (2230R1r) to 3.57 (227bR1r) for the anxiety item bank and ranging from 1.74 (5035R1r) to 4.36 (5035R1r) for the depressive symptoms item bank. Threshold parameters ranged from − 0.47 to 3.18 and − 1.24 to 2.04 for the anxiety and depressive symptoms item banks, respectively. In both item banks, all items had non-significant X^2^ values, indicating that the items fit the model.

**Table 2:** Item parameters and item fit statistics for the Swedish pediatric Patient-Reported Outcomes Measurement Information System (PROMIS) Anxiety and Depressive symptoms item banks

	Item parameters					Item fit statistics		
	a	b_1_	b_2_	b_3_	b_4_	S − X^2^	df	p − S − X^2^
*Anxiety*								
2220R2r	2.67	0.40	0.94	1.64	2.21	89.22	72	0.08
713R1r	3.43	− 0.47	0.15	0.84	1.55	92.98	67	0.02
227bR1r	3.57	0.17	0.79	1.51	2.06	68.56	58	0.16
5044R1r	2.80	− 0.45	0.20	1.13	1.86	70.01	69	0.44
3459bR1r	2.43	0.45	1.11	1.71	2.38	72.42	74	0.53
2230R1r	1.49	0.36	1.15	1.83	2.43	99.82	105	0.62
231R1r	2.70	0.36	0.97	1.66	2.24	85.01	69	0.09
3150bR2r	2.73	0.04	0.53	1.21	1.76	99.46	84	0.12
7005	2.05	0.17	0.80	1.44	1.97	95.74	95	0.46
3021R1r	1.88	0.95	1.66	2.39	3.18	50.86	61	0.82
3149R1r	2.18	0.86	1.36	2.05	2.69	75.76	71	0.33
3459aR1r	2.74	0.50	1.05	1.65	2.24	63.26	69	0.67
3977R1r	2.38	0.72	1.21	1.68	2.06	82.91	77	0.30
7006	1.90	0.30	1.00	1.66	2.37	113.15	89	0.04
953R1r	2.18	− 0.43	0.24	0.98	1.78	81.47	88	0.67
*Depressive symptoms*								
488R1r	3.45	0.05	0.64	1.40	1.96	81.12	66	0.10
461R1r	3.60	− 0.03	0.45	1.11	1.68	58.55	66	0.73
5041R1r	4.17	0.05	0.52	1.06	1.64	65.03	59	0.27
5035R1r	3.95	− 0.10	0.44	1.05	1.59	54.33	61	0.71
711R1r	3.51	− 0.26	0.28	0.96	1.56	49.76	72	0.99
228R1r	4.30	− 0.50	0.11	0.93	1.55	59.09	55	0.33
712R1r	4.36	0.07	0.63	1.31	1.78	49.89	60	0.82
3952aR2r	3.26	− 0.25	0.46	1.15	1.91	46.53	68	0.98
2227R1r	2.63	− 0.03	0.60	1.32	1.91	112.71	88	0.04
2697R1r	1.78	− 0.90	− 0.12	0.92	1.82	108.47	99	0.24
5047R1r	1.74	− 1.24	− 0.51	0.49	1.38	86.86	102	0.86
679aR2r	3.22	0.41	1.00	1.58	2.04	55.27	60	0.65
7010	3.51	0.03	0.50	1.08	1.69	83.11	71	0.15
9001r	2.88	0.58	1.02	1.56	2.03	59.60	71	0.83

a: Discrimination parameter, b_1_–b_4_: Threshold values, S − X^2^: Orlando and Thissen’s S − X^2^ Statistics, df: Degrees of freedom, p − X^2^: Significance value for the X^2^

No DIF was found for sex (girls, boys), age groups (12–15, 16–20 years) or type (school, patient sample) for the anxiety or depressive symptoms item banks. Language DIF (U.S. vs. Swedish items) was found for two item from the anxiety item bank and one item from the depressive symptoms item bank. Uniform DIF was found language for item 2220R2r, “I felt like something awful might happen” (R^2^ = 0.03) and 231R1r, “I worried about what could happen to me” (R^2^ = 0.07) from the anxiety item bank, and item 712R1r, "I felt unhappy" (R^2^ = 0.06) from the depressive symptoms item bank.

The mean T-scores for the Swedish sample on the official U.S. PROMIS T-score metric were 48.10 (SD = 11.82) for the anxiety item bank and 51.21 (SD = 12.91) for the depressive symptom item bank.

Known-group analysis compared Swedish T-scores between CAP and school samples. An independent sample t-test showed a significant difference between the CAP and the school sample for both item banks. Estimated mean T-score of 55.0 in the CAP group and 47.7 in the school sample (t = − 10.49, df = 457.49, p < 0.001) for the anxiety item bank and 54.7 in the CAP sample and 47.9 in the school sample (t = − 10.057, df = 506.93, p < 0.001) for the depressive symptoms item bank. The effect size was large to medium with Cohen’s d − 0.81, 95% CI [− 0.96, − 0.66], and − 0.74 [− 0.89, − 0.60] for the anxiety and depressive symptoms item banks, respectively. Thus, evidence for known-group validity between the CAP and school sample for both item banks was given. For the anxiety item bank the percentage of floor effects was 17. 06% and 7.78% and ceiling effects 0% and 0.37% for the school and CAP samples, respectively. For the depressive symptoms item bank the percentage of floor effects was 11. 65% and 3.70% and ceiling effects 0.17% and 0.74% for the school and CAP samples, respectively.

Table 3 presents the post-hoc CAT estimates for the anxiety and depressive symptoms item banks, respectively. The CAT algorithm sorts data into ten equal parts/deciles (D1–D10), with the sample equally divided between the deciles each decile. Each decile provides a mean theta score and the corresponding, e.g., mean standard error, mean bias, RMSE, and mean test length. We adopted the hybrid approach with U.S. parameters for the second post-hoc CAT simulation with U.S. parameters for all items except the DIF language items, where Stocking-Lord transformed Swedish item parameters were used [38]. See supplementary table for Stocking Lord constants for the anxiety and depressive symptoms item bank and parameter estimates.

**Table 3:** Computer adaptive testing simulations for the Swedish pediatric Patient-Reported Outcomes Measurement Information System (PROMIS) Anxiety and Depressive symptoms item banks with Swedish and US parameters

Anxiety	D1	D2	D3	D4	D5	D6	D7	D8	D9	D10
*Swedish parameters* Mean test length 7.43 Mean RMSE 0.22 Mean bias -0.01										
Mean Theta	–	− 1.42	− 0.81	− 0.50	− 0.18	0.15	0.34	0.54	0.89	1.48
RMSE	–	0.001	0.05	0.19	0.21	0.18	0.29	0.32	0.30	0.33
Mean bias	–	0.001	0.02	0.12	0.04	0.03	0.04	− 0.09	− 0.14	− 0.10
Mean test length	–	15	13.9	7.05	4.75	4.3	3.58	3.47	3.5	3.7
Mean SE	–	0.52	0.34	0.30	0.30	0.30	0.30	0.30	0.30	0.30
Proportion stop rule satisfied	–	0	0.2	1	1	1	1	1	1	1
Number of simulees	–	39	20	19	20	20	19	19	20	20
Reliability	–	0.73	0.88	0.91	0.91	0.91	0.91	0.91	0.91	0.91
*US parameters* Mean test length 13.39 Mean RMSE 0.27 Mean bias -0.10										
Mean Theta	–	− 1.42	− 0.81	− 0.50	− 0.18	0.15	0.34	0.54	0.89	1.49
RMSE	–	0.29	0.37	0.33	0.21	0.11	0.12	0.13	0.218	0.43
Mean bias	–	− 0.29	− 0.36	− 0.31	− 0.20	− 0.05	− 0.04	0.05	0.12	0.37
Mean test length	–	15	15	15	15	13.65	12.47	11.84	10.5	10.5
Mean SE	–	0.56	0.46	0.39	0.34	0.32	0.32	0.31	0.31	0.31
Proportion stop rule satisfied	–	0	0	0	0.05	0.45	0.68	0.84	0.95	0.90
Number of simulees	–	39	20	19	20	20	19	19	20	20
Reliability	–	0.68	0.79	0.84	0.88	0.90	0.90	0.90	0.90	0.90
Depressive symptoms	D1	D2	D3	D4	D5	D6	D7	D8	D9	D10
*Swedish parameters* Mean test length 5.27 Mean RMSE 0.26 Mean bias -0.01										
Mean Theta	–	− 1.45	− 0.73	− 0.45	− 0.24	0.14	0.39	0.68	0.93	1.47
RMSE	–	0.02	0.21	0.22	0.22	0.33	0.28	0.29	0.38	0.32
Mean bias	–	0.01	0.15	0.15	0.05	0.09	− 0.12	− 0.15	− 0.22	− 0.05
Mean test length	–	13.65	5.85	3.65	3.05	2.60	2.30	2.39	2.36	3.1
Mean SE	–	0.48	0.30	0.28	0.29	0.29	0.29	0.28	0.29	0.30
Proportion stop rule satisfied	–	0.05	1	1	1	1	1	1	1	0.95
Number of simulees	–	40	20	20	19	20	20	18	22	20
Reliability	–	0.77	0.91	0.92	0.92	0.92	0.92	0.92	0.92	0.91
*US parameters* Mean test length 9.73 Mean RMSE 0.39 Mean bias 0.26										
Mean Theta	–	− 1.45	− 0.73	− 0.45	− 0.24	0.14	0.39	0.68	0.93	1.47
RMSE	–	0.13	0.11	0.22	0.32	0.43	0.43	0.50	0.55	0.67
Mean bias	–	− 0.10	− 0.04	0.17	0.27	0.39	0.40	0.44	0.50	0.64
Mean test length	–	14	14	12.65	9.79	7.20	6.65	6.22	5.95	6.9
Mean SE	–	0.53	0.38	0.32	0.31	0.31	0.31	0.31	0.31	0.31
Proportion stop rule satisfied	–	0	0	0.5	0.95	1	1	0.94	1	1
Number of simulees	–	40	20	20	19	20	20	18	22	20
Reliability	–	0.72	0.85	0.90	0.91	0.91	0.91	0.91	0.91	0.91

D1-D10: deciles 1–10. The decils divides the CAT into 10 equal parts with approximately 20 respondents each. Theta: underlying ability of the construct, *RMSE* Root Mean Square Error, test length: Items administered, *SE* Standard Error. Reliability = 1 − SE^2^

The mean test length for the anxiety item bank for the Swedish and U.S. parameters was 7.43 and 13.39 items, the mean RMSE was 0.22 and 0.27, and the mean bias was − 0.01 and − 0.1. The post-hoc with U.S. parameters needed more items than the Swedish to obtain the stopping rule of a standard error of 0.32; the mean bias and mean bias per decile are generally low in both item banks; however, they are highest in D10 for the U.S. parameters at the highest theta scores, an indication that the CAT overestimates the ability at this end of the latent trait. The difference in mean RMSE is negligible and reflects overall low error values. The correlation between theta from the post-hoc CAT and the full item bank was high, 0.97 for Swedish and 0.99 for U.S. parameters. The proportion of simulees that satisfied the stop criterion was 0.72 for Swedish and 0.39 for U.S. parameters.

For the depressive symptoms item bank, for the Swedish and U.S. parameters, the mean test length was lower between 5.27 and 9.73 items, the mean RMSE was slightly higher, 0.26 and 0.39, and the mean bias was identical for the Swedish − 0.01 and higher for the U.S. parameters 0.26. The mean bias is overall higher on the U.S. parameters, especially at higher levels of theta, with the highest mean bias at D10; the same pattern is seen with RMSE. The correlation between theta from the post-hoc CAT and the theta and the full item bank was high, 0.96 and 0.99 for Swedish and U.S. parameters, respectively. The proportion of simulees that satisfied the stop criterion was 0.80 for Swedish and 0.63 for U.S. parameters.

**Fig. 1:**
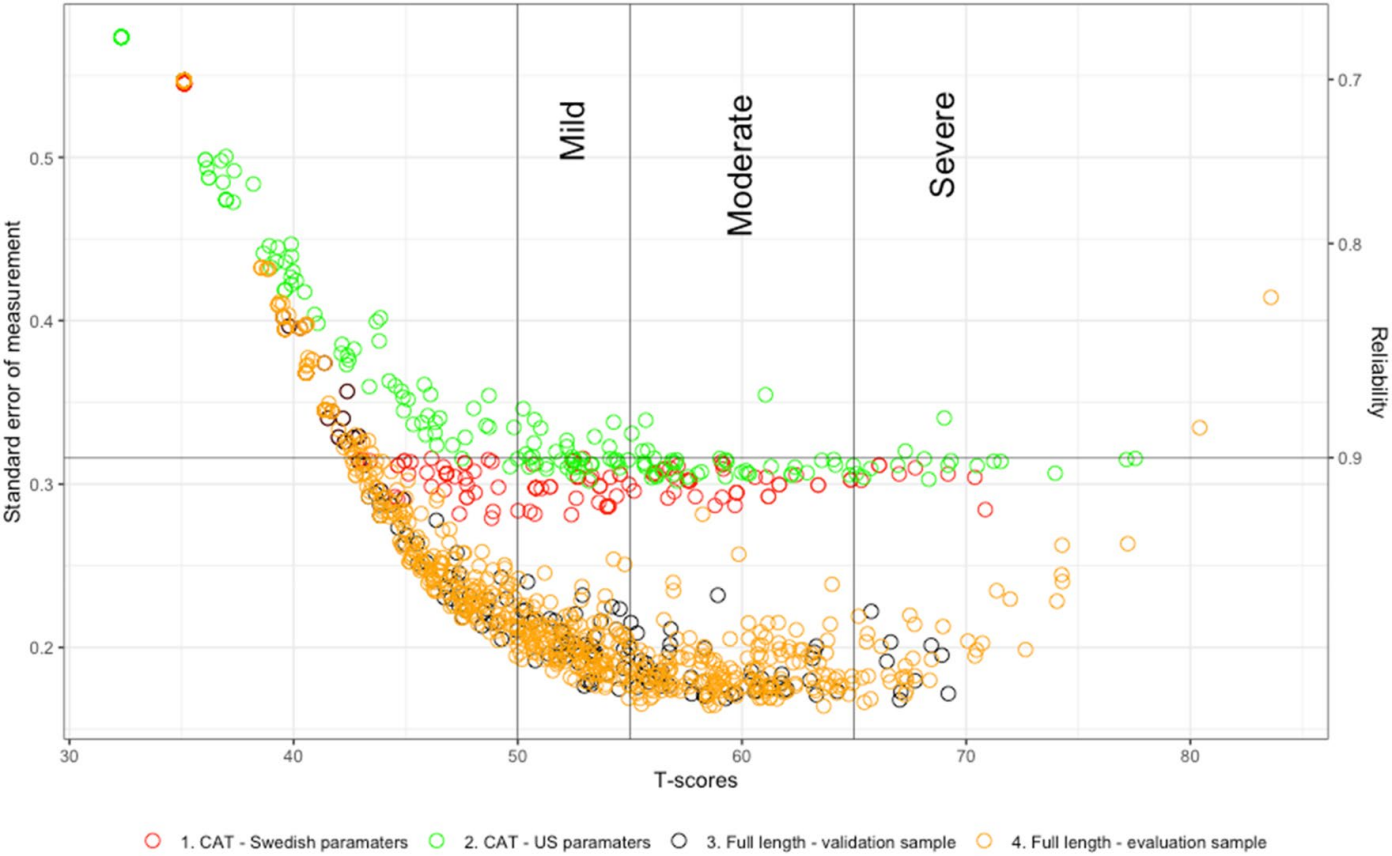
Reliability of the Swedish pediatric PROMIS Anxiety item bank comparing CAT simulation with Swedish parameters on the validation sample, CAT simulation with US parameters on the validations sample with full length item bank on the validation and evaluation samples

**Fig. 2:**
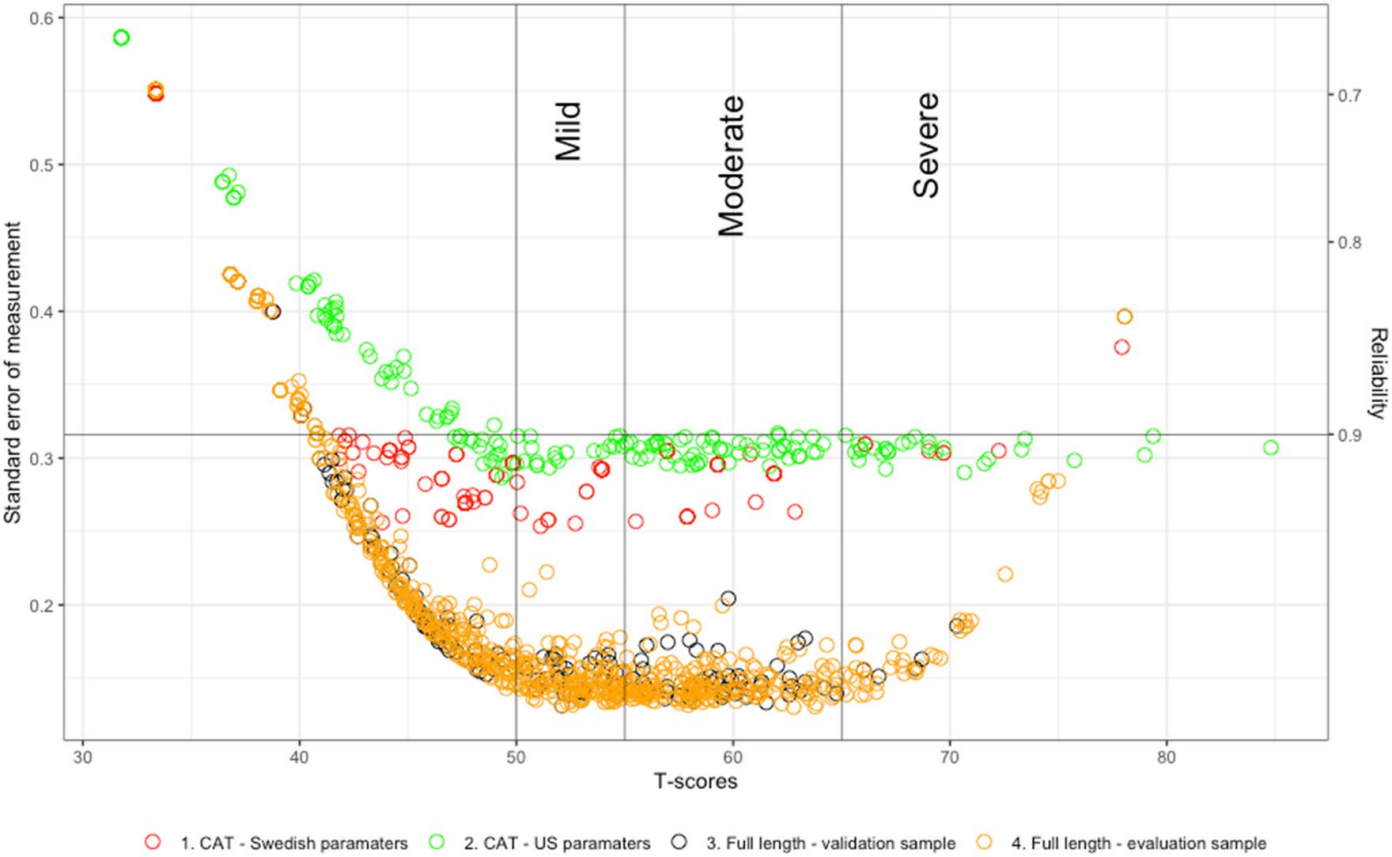
Reliability of the Swedish pediatric PROMIS Depressive symptoms item bank comparing CAT simulation with Swedish parameters on the validation sample, CAT simulation with US parameters on the validations sample with full length item bank on the validation and evaluation samples

Figures 1 and 2 show the post-hoc CAT simulations with the standard error of measurement and the corresponding reliability on the Y-axis and T-scores on the X-axis. The figure compares the post-hoc CAT with Swedish parameters, U.S. parameters, and full item banks concerning T-score distribution and reliability. The two post-hoc CAT simulations (red dots—post-hoc CAT on validation sample with Swedish parameters and green dots—post-hoc CAT on validation sample with U.S. parameters) compared with the full item banks (black dots—Full item bank validation sample and yellow dots—Full item bank evaluation sample). The figures also display the interpretation of PROMIS T-scores for the anxiety and depressive symptoms item banks from HealthMeasures (HealthMeasures., PROMIS Score Cut Points [40]); T-scores of < 50 are interpreted as within normal limits, 50–55 mild, 55–65 moderate, and > 65 severe anxiety or depressive symptoms.

## Discussion

This study validated the Swedish pediatric PROMIS item banks of anxiety and depressive symptoms in a Swedish school- and CAP patient sample. Sufficient unidimensionality, local independence, and monotonicity were found. The IRT model fit was acceptable for both item banks. We found no DIF for sex, age groups, or sample type. Language DIF (Swedish vs. U.S) was found for two items from the anxiety item bank and one item from the depressive symptoms item bank. Post-hoc CAT was performed separately with Swedish and official U.S. parameters to conform with PROMIS practice. We found reliability to be above 0.84 in the range of within normal limits to severe anxiety and depressive symptoms for Swedish and US parameter post-hoc CAT simulations and both item banks. The post-hoc simulations showed a high correlation between the full and CAT item banks.

In the CFA, RMSEA exceeded the cut-off, consistent with findings from previous PROMIS item bank studies [11, 12, 41, 42]. SRMR, a more arguable suitable fit index for ordinal data, was within the cut-off, as were the other fit indices, supporting acceptable model fit [39]. Additionally, KMO, parallel analysis, and EFA confirmed unidimensionality.

The PROMIS anxiety and depressive symptoms item banks have been validated in a Dutch general sample, where lower T-scores were found compared to a U.S. representative sample [11]. The T-scores for our sample were more similar to those of the U.S. representative sample than the Dutch general sample [11], with T-scores slightly lower (the anxiety item bank) and slightly higher (the depressive symptoms item bank). Since our sample, apart from school students, had Child and Adolescent Psychiatry patients, higher T-scores were expected. Both the anxiety and depressive symptoms item banks possess satisfactory known-group validity regarding sample type; CAP, or school sample. For the anxiety item bank T-scores in the school sample, the floor effect was 17.06%, slightly above the cut-off, which is unsurprising considering it is a general sample expected to have lower values. In the CAP sample for the anxiety item bank, the floor effect was 7.78%; hence, in the combined sample of CAP and school respondents, the overall flooring effect was below 15%. All other floor/ceiling effects were under the cut-off.

For both item banks, the lowest decile (D2) needed all items to be administered with a reliability of between 0.68 to 0.73 (anxiety item bank, and 0.77 to 0.72 (depressive symptoms item bank) and with a T-score of approximately 36, reflecting within normal levels of symptoms. The reliability increases in the following deciles with gradually higher average thetas (i.e., T-scores), and the number of items administered decreases. Hence, precise measurement with significant item reduction is demonstrated, which is further emphasized by the high correlation between the full item bank and post- hoc CAT (> 0.98) for both banks. Overall, the bias and RMSE are low, indicating a precise measurement without significant over- or underfitting of the estimated and true thetas. At the highest end of the thetas scores (D10), i.e., at the severest symptoms burden, the RMSE and bias are slightly higher in the post-hoc CAT with the U.S. item parameters. This suggests that the post-hoc CAT overestimates ability levels and less item coverage, emphasizing the need for more items. A possible explanation is that our sample consists of CAP patients with higher mean T-scores than the U.S. reference sample. In the future, it would be preferable to include more items with higher symptom severity to enhance the scale further. Even though the Swedish item parameters performed better than the hybrid U.S. parameters, the overall conclusion is to use the Universal T-score metric to enhance the possibility of comparing symptom burdens between countries. However, an additional future direction would be to broaden the Universal T-score metric with CAP samples and perhaps also scores from more countries.

A limitation of this study was that we lacked appropriate ways to evaluate whether the respondents answered truthfully. They could have answered entirely at random without consideration of the item or their actual feelings. Such respondents can be hard to detect. One possible way of detecting them is by adding reverse-coded items and deleting those with conflicting answers [11]. Also, due to the GDPR jurisdiction and the consequent online platform updates required, we collected some of the data on paper. However, research on other scales has not shown significant differences in whether the data was adhered to online or in paper format [11]. Sample planning was based on recommendations [20, 43] rather than a priori power calculations; therefore, results should be interpreted with caution.

In conclusion, the Swedish PROMIS pediatric item banks of anxiety and depressive symptoms showed acceptable unidimensionality, local independence, and monotonicity. The subsequent GRM showed adequate item fit for all items. No DIF was found for sex, age groups, or type of sample. Known-group validity was shown for the differentiation between CAP and school sample for both item banks. Further, both item banks had excellent reliability from mild to severe levels of anxiety and depressive symptoms. Post-hoc computer adaptive testing (CAT) simulations indicate that the item banks are appropriate for CAT. To further enhance the settings for CAT, a future research direction is to validate additional items especially, at the severest symptoms level. The next step will be the implementation of the Swedish PROMIS item banks of anxiety and depressive symptoms in CAP clinics as a clinical screening tool.

## Supplementary Information

Below is the link to the electronic supplementary material.

Supplementary file1 (DOCX 36 kb)

## Data Availability

The corresponding author may provide the datasets used and analyzed during the current study on reasonable request.
