# Supplementary material for: Psychometric properties and post-hoc CAT analysis of the pediatric PROMIS® item banks anxiety and depressive symptoms in a combined Swedish Child and Adolescent Psychiatry and School sample
Source: Qual Life Res. 2025 Jan 30;34(5):1265–75. doi: 10.1007/s11136-025-03898-y (PMC12064460; doi:10.1007/s11136-025-03898-y)
Supplement: Supplementary file 1 — Supplementary file1 (DOCX 36 kb) [file 11136_2025_3898_MOESM1_ESM.docx]

# Supplementary tables: Item response theory validation of the Swedish pediatric PROMIS item banks of anxiety and depressive symptoms in clinical and community samples

Table A. Stocking Lord constants pediatric Patient-Reported Outcomes Measurement Information System (PROMIS) Anxiety item bank

| **Stocking Lord constant** |  |
| --- | --- |
| A | 1.45 |
| B | −0.16 |

Table B. Stocking-Lord equated Swedish parameters – pediatric Patient-Reported Outcomes Measurement Information System (PROMIS) Anxiety item bank

|  | **a** | **b1** | **b2** | **b3** | **b4** |
| --- | --- | --- | --- | --- | --- |
| **PROWORRY1** | 1.8 | 0.43 | 1.21 | 2.23 | 3.05 |
| **PROWORRY2** | 2.36 | −0.84 | 0.06 | 1.07 | 2.10 |
| **PROWORRY3** | 2.46 | 0.09 | 0.99 | 2.04 | 2.84 |
| **PROWORRY4** | 1.93 | −0.81 | 0.14 | 1.48 | 2.55 |
| **PROWORRY5** | 1.67 | 0.49 | 1.46 | 2.32 | 3.31 |
| **PROWORRY6** | 1.03 | 0.37 | 1.52 | 2.50 | 3.38 |
| **PROWORRY8** | 1.86 | 0.37 | 1.25 | 2.26 | 3.10 |
| **PROWORRY10** | 1.88 | −0.10 | 0.61 | 1.61 | 2.41 |
| **PROWORRY11** | 1.41 | 0.09 | 1.01 | 1.94 | 2.71 |
| **PROWORRY12** | 1.29 | 1.22 | 2.26 | 3.32 | 4.47 |
| **PROWORRY13** | 1.50 | 1.09 | 1.82 | 2.83 | 3.75 |
| **PROWORRY14** | 1.88 | 0.56 | 1.37 | 2.24 | 3.10 |
| **PROWORRY15** | 1.64 | 0.88 | 1.60 | 2.29 | 2.84 |

Table C. Stocking Lord constants pediatric Patient-Reported Outcomes Measurement Information System (PROMIS) Depressive symptoms item bank

| **Stocking Lord constant** |  |
| --- | --- |
| A | 1.56 |
| B | 0.12 |

Table D. Stocking-Lord equated Swedish parameters – pediatric Patient-Reported Outcomes Measurement Information System (PROMIS) Depressive symptoms item bank

|  | **a** | **b1** | **b2** | **b3** | **b4** |
| --- | --- | --- | --- | --- | --- |
| **PROD1** | 2.23 | 0.21 | 1.13 | 2.31 | 3.19 |
| **PROD2** | 2.30 | 0.07 | 0.83 | 1.86 | 2.76 |
| **PROD3** | 2.67 | 0.20 | 0.93 | 1.78 | 2.68 |
| **PROD4** | 2.52 | −0.03 | 0.82 | 1.77 | 2.61 |
| **PROD5** | 2.24 | −0.29 | 6 | 1.63 | 2.56 |
| **PROD6** | 2.75 | −0.66 | 0.30 | 1.58 | 2.55 |
| **PROD7** | 2.79 | 0.23 | 1.12 | 2.18 | 2.91 |
| **PROD8** | 2.09 | −0.27 | 0.84 | 1.92 | 3.10 |
| **PROD9** | 1.68 | 0.08 | 1.07 | 2.20 | 3.11 |
| **PROD10** | 1.14 | −1.29 | −0.07 | 1.56 | 2.97 |
| **PROD11** | 1.11 | −1.81 | −0.68 | 0.88 | 2.28 |
| **PROD12** | 2.06 | 0.76 | 1.69 | 2.60 | 3.32 |
| **PROD13** | 2.25 | 0.17 | 0.91 | 1.81 | 2.76 |
| **PROD14** | 1.84 | 1.03 | 1.72 | 2.56 | 3.29 |
